# A Synthesis of Hepatitis C prevalence estimates in Sub-Saharan Africa: 2000–2013

**DOI:** 10.1186/s12879-016-1584-1

**Published:** 2016-06-13

**Authors:** Nallely Mora, William H. Adams, Stephanie Kliethermes, Lara Dugas, Neelam Balasubramanian, Jasmin Sandhu, Helen Nde, Christina Small, Joanne Jose, Steven Scaglione, Jennifer E. Layden

**Affiliations:** Departments of Medicine and Public Health Sciences, Loyola Stritch School of Medicine, 2160 S. First Ave., Maywood, IL 60153 USA; Department of Medicine, Loyola Stritch School of Medicine, 2160 S. First Ave., Maywood, IL 60153 USA; Loyola University Chicago Health Science Campus, Fahey Building Room 116, 2160 S. First Ave., Maywood, IL 60153 USA

**Keywords:** HCV, Hepatitis, Prevalence, Meta-analysis, Africa, Sub-Saharan

## Abstract

**Background:**

Hepatitis C (HCV) is a deleterious virus that can be cured with new, highly effective anti-viral treatments, yet more than 185 million individuals worldwide remain HCV positive (with the vast majority un-diagnosed or untreated). Of importance, HCV is a leading cause of chronic liver disease and liver cancer, especially in Sub-Saharan Africa (SSA) where the prevalence remains high but uncertain due to little population-based evidence of the epidemic. We aimed to synthesize available data to calculate and highlight the HCV disease burden in SSA.

**Methods:**

Weighted random-effects generalized linear mixed models were used to estimate prevalence by risk cohort, African region (Southern, Eastern, Western, and Central Africa), type of assay used, publication year, and whether the estimate included children. A pooled prevalence estimate was also calculated. Multi-variable analyses were limited to cohort and region specific prevalence estimates in the adult population due to limited studies including children. Prevalence estimates were additionally weighted using the known adult population size within each region.

**Results:**

We included more than 10 years of data. Almost half of the studies on HCV prevalence in SSA were from the Western region (49 %), and over half of all studies were from either blood donor (25 %) or general population cohorts (31 %). In uni-variable analyses, prevalence was lowest in Southern Africa (0.72 %), followed by Eastern Africa at 3.00 %, Western Africa at 4.14 %, and Central Africa at 7.82 %. Blood donors consistently had the lowest prevalence (1.78 %), followed by pregnant women (2.51 %), individuals with comorbid HIV (3.57 %), individuals from the general population (5.41 %), those with a chronic illness (7.99 %), and those at high risk for infection (10.18 %). After adjusting for the population size in each region, the overall adult prevalence of HCV in SSA rose from 3.82 to 3.94 %.

**Conclusion:**

This meta-analysis offers a timely update to the HCV disease burden in SSA and offers additional evidence of the burgeoning epidemic. The study highlights the need to account for type of cohort and region variation when describing the HCV epidemic in SSA, the need for more studies that include children, as well as the need to factor in such variations when planning public health interventions.

**Electronic supplementary material:**

The online version of this article (doi:10.1186/s12879-016-1584-1) contains supplementary material, which is available to authorized users.

## Background

Hepatitis C Virus (HCV) is a leading cause of chronic liver disease, cirrhosis, and liver cancer worldwide and is a primary indication for liver transplantation [1]. With no available vaccine, treatment and disease prevention remain the primary methods to reduce disease burden. Fortunately, new highly active anti-viral agents provide the opportunity to cure diagnosed and treated individuals [2]. As such, a renewed interest in estimating the global disease burden of this deadly infection is both timely and necessary.

Significant discrepancies exist in recent HCV global burden estimates. Mohd Hanafiah et al. [3] estimated >185 million individuals worldwide were HCV antibody positive (sero-positive) in 2005, a 52 % increase from 1990 and, recently, Gower et al. (2014) estimated 115 million individuals world-wide were sero-positive, noting more than 80 million individuals expressed viremic infection [4]. Discrepancies in these estimates are partially due to applied inclusion and exclusion criteria, particularly whether estimates were derived using blood donors and minors as well as the time periods that capture available data.

Despite these differences, both studies reveal that Sub-Saharan Africa (SSA) suffers from an alarming HCV disease burden. Our true understanding of the epidemic in SSA is unfortunately profoundly limited by a lack of robust primary data, including limited population-based studies [4]. As such, there is significant uncertainty when estimating the true disease burden in SSA. A recent meta-analysis revealed a pooled HCV sero-prevalence of 2.98 % across SSA [5], and studies have identified significant variability across geographic region (i.e., Central, Southeastern, and Western Africa) and risk group (e.g., blood donors, pregnant women, those with an existing liver disease, substance users, etc.) [3, 5]. Such variation suggests an overall disease burden estimate may not be appropriate for SSA. Previous work also suggests that the serologic assay used to determine HCV status may impact prevalence estimates due to variation in false positivity rates [6–8]. Further, it is speculated that children have lower overall disease burden, and estimates that include or exclude children vary considerably in previously reported estimates [9]. Consequently, it is important for prevalence estimates to account for geographic region of SSA, study population, year of study, and diagnostic assay used, because these parameters may greatly impact disease estimates. Rarely have studies adjusted prevalence estimates to account for these effect modifiers, nor have studies attempted to adjust estimates based on the composition of the population in general.

The primary aim of this study was to conduct a meta-analysis on the HCV sero-prevalence in SSA, examining the impact of region, cohort, year of study, and diagnostic assay used to generate the estimates. A secondary aim used the model based cohort estimates to derive a weighted prevalence based on the population sizes in Western Africa.

## Methods

### Literature search

This study adhered to the meta-analysis of observational studies (MOOSE) guidelines established in 2008 [10]. Articles and abstracts were identified for this meta-analysis by searching the following academic search engines: (1) Medline, (2) Ovid, (3) EMBASE, (4) Google Scholar, (5) PubMed, and (6) Academic Search Complete/EBSCO using keywords (a) “Hepatitis C AND Sub-Saharan Africa”, (b) “HCV AND central Africa”, (c) “HCV AND eastern Africa”, (d) “HCV AND western Africa”, (e) “HCV AND southern Africa” and (f) “HCV AND prevalence AND Africa”. When articles or abstracts were identified, snowball sampling was employed to find additional articles referenced in the published material.

We limited data capture to articles published from January 2000 through December 2013 that sampled from mainland countries in SSA. Only publications printed in English, Spanish, and French were retained. Articles without original data or without HCV prevalence estimates were excluded.

### Data capture

All studies were randomly distributed using a random numbers table among study team members (NM, NB, JS, HN, and CS) for double data entry. An electronic query system flagged discrepancies which two statisticians (SK and WA) reviewed and resolved. HCV prevalence estimates and the absolute numbers used to generate such estimates were recorded for each study. Studies were grouped into one of four Sub-Saharan regions using boundaries described by the World Health Organization, namely Central, Eastern, Western, or Southern Africa [11]. Publication year, the highest generation diagnostic assay used (i.e., screening, second, third, or fourth generation assay), and whether studies enrolled individuals under age 16 were also recorded.

Each sample was additionally grouped into a population cohort representing either (1) blood donors, (2) those at high risk for infection (e.g., studies of prisoners and prison guards, adults and children with sickle cell disease, hospital workers, sex workers, intravenous drug users, and hemodialysis patients), (3) individuals with comorbid HIV infection, (4) those with a chronic Illness (e.g., individuals with diabetes, those with a chronic liver disease, or patients admitted to a healthcare facility), or (5) pregnant women; studies of household members, adults, outpatients, healthy children, and infants were grouped into a fifth category representing the general population.

### Statistical analyses

All analyses were conducted using SAS software version 9.4, (SAS Institutes, Cary, NC). Prevalence estimates were weighted using the inverse of the summation of their between and within study variances (as described by Hedges and Veva, 1998) [12]. Weighted random-effects binomial generalized linear mixed models with logit links were used to estimate prevalence within population cohorts, SSA regions, type of assay used, publication year, and inclusion of minors. In these univariable models, each moderator (i.e., population cohort, region, type of assay used, publication year, and whether minors were included) represented a fixed effect while random intercepts were allowed for each study contributing estimates. To determine a *total* population prevalence estimate of Hepatitis C in SSA, an intercept only weighted random-effects binomial model was used, allowing for random intercepts among studies.

Statistical interactions were also assessed using multivariable random-effects models. For example, a weighted binomial random-effects model was used to further assess a significant region-by-cohort interaction. In this model, region, cohort, and their interaction were fixed effects while random intercepts were allowed for each study contributing estimates. Studies that included minors under age 16 (k = 18) were excluded and, as such, these multivariable results report Hepatitis C prevalence estimates only for adults.

We next derived estimates of HCV disease burden that accounted for adult population sizes within each region. The 2015 United Nations population database was used to provide the overall population size in each region for individuals ≥ 15 years old [13]. The estimated number of anti-HCV *adult* individuals per region was calculated by multiplying each model based region prevalence estimate by the number of adults within that region. A pooled prevalence estimate was derived that was weighted by the population size within each region.

## Results

### Composition of HCV Sero-prevalence studies in Sub-Saharan Africa

**Fig. 1:**
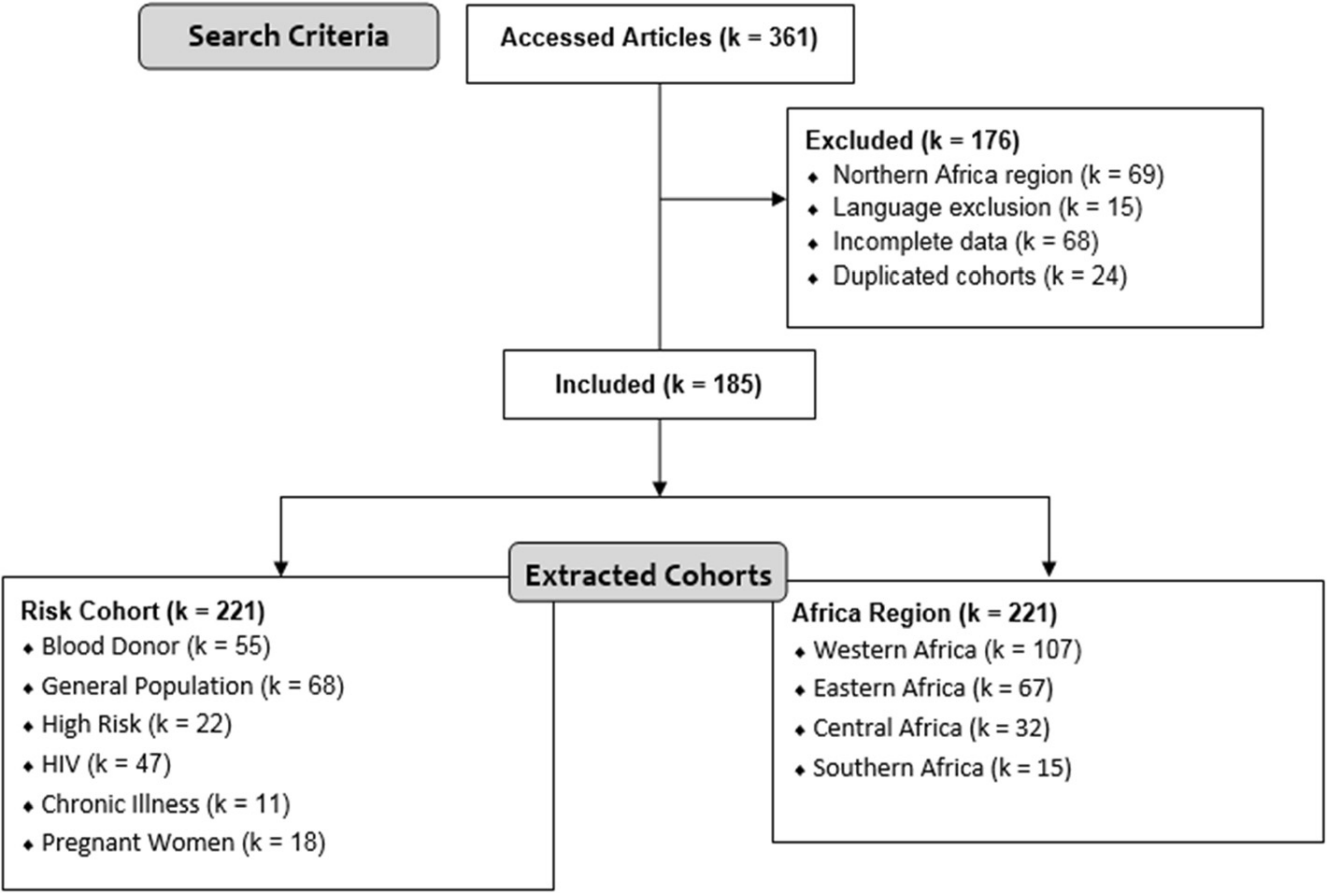
Article selection and cohort identification

Of the 361 published articles and abstracts identified for this analysis 185 articles comprising K = 221 *independent* cohorts were retained (i.e., articles were allowed to contribute multiple independent prevalence estimates - see Additional file 1: Table S1). Primary reasons for the exclusion of articles were that they sampled from WHO defined Northern Africa (k = 69), were printed in a language other than English, French, or Spanish (k = 15), had incomplete data (k = 68) or were repeat studies that engendered dependency (k = 24). See Fig. 1.

Figure 4 displays the number of articles representing Western Africa populations (K = 107; 48.5 % of all articles), Eastern Africa (K = 67; 30.3 %), Central Africa (K = 32; 14.4 %), and Southern Africa (K = 15; 6.8 %) populations. The distribution of studies by cohort and region excluding studies that had children is shown in Fig. 5; studies including children were excluded for this because subsequent meta-analyses excluded these studies. Across all regions, the largest proportion of studies reporting prevalence estimates occurred among the general population and blood donors (27.1 % each), followed by HIV infected individuals (21.7 %). However, there was dramatic variation in the distribution of cohorts with prevalence estimates by region, as seen in Fig. 5. For example, 64.3 % of all studies conducted in Southern Africa were among HIV infected individuals. In comparison, HIV cohorts made up 18.3, 25.4, and 3.8 % of studies, respectively, in Western, Eastern, and Central Regions.

### Uni-variable prevalence estimates

**Fig. 2:**
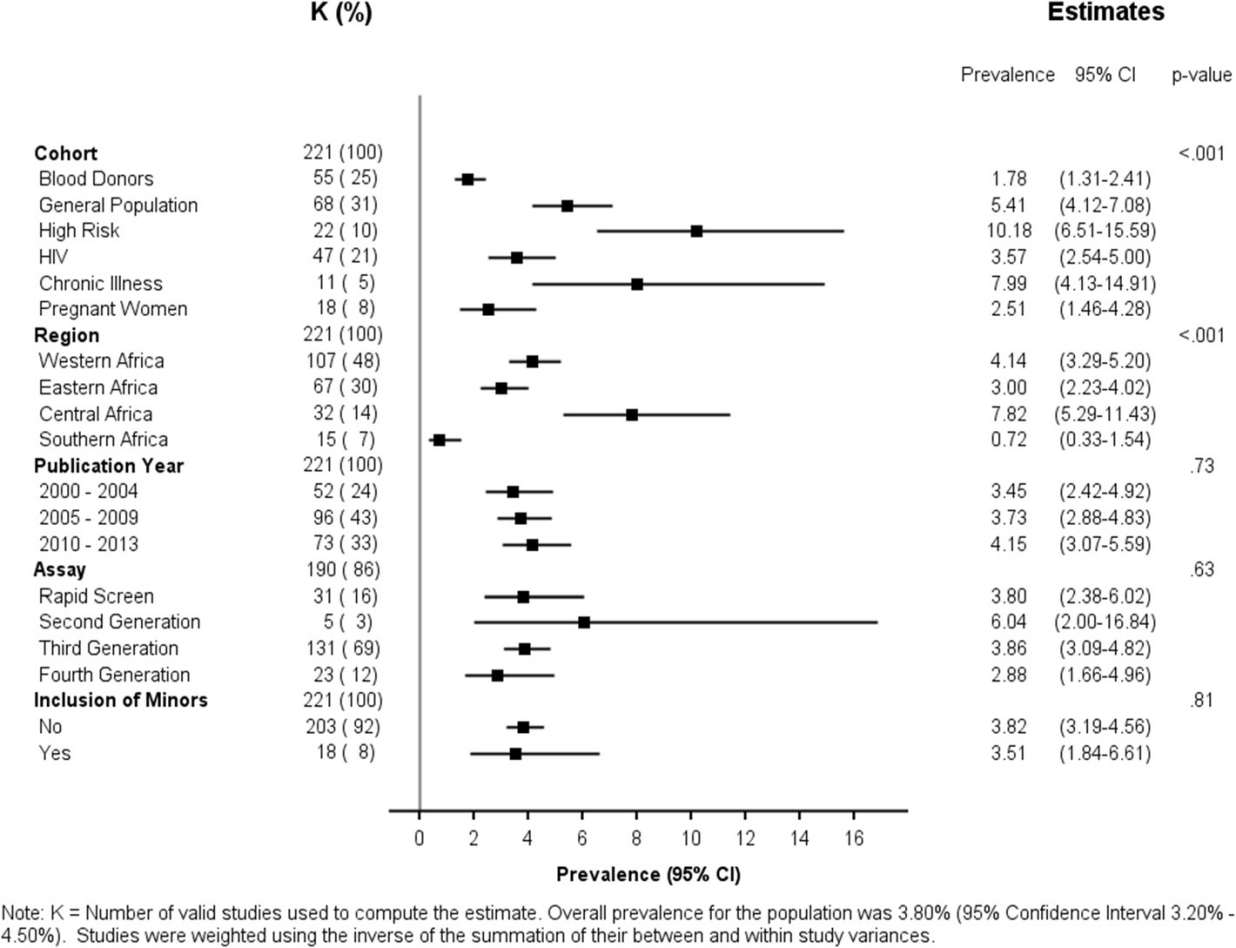
Univariable seroprevalence estimates of HCV infection by moderator

The precision of the prevalence estimates by cohort and other parameters are shown in Fig. 2. While prevalence estimates significantly varied across African regions and cohorts (both *p* < .001), there was no significant variability between the inclusion and exclusion of minors (*p* = .81), nor by generation of diagnostic assay (*p* = .63) or publication year (*p* = .73). Prevalence in Southern Africa was low - estimated at 0.72 % (95 % CI: 0.33–1.54 %). This was followed by Eastern Africa estimated at 3.00 % (95 % CI: 2.23–4.02 %) and Western Africa estimated at 4.14 % (95 % CI: 3.29–5.20 %); Central Africa (7.82 %, 95 % CI: 5.29–11.43 %) had the highest estimated prevalence of Hepatitis C.

Among the six population cohorts, blood donors had the lowest prevalence (1.78 %, 95 % CI: 1.31–2.41 %) followed by pregnant women (2.51 %, 95 % CI: 1.46–4.28), individuals with HIV (3.57 %, 95 % CI: 2.54–5.00 %) and those from the general population (5.41 %, 95 % CI: 4.12–7.08 %). Higher prevalence was observed for both individuals with a chronic illness (7.99 %, 95 % CI: 4.13–14.91 %) and those considered at high risk for infection (10.18 %, 95 % CI: 6.51–15.59 %).

### Multivariable prevalence estimates

**Fig. 3:**
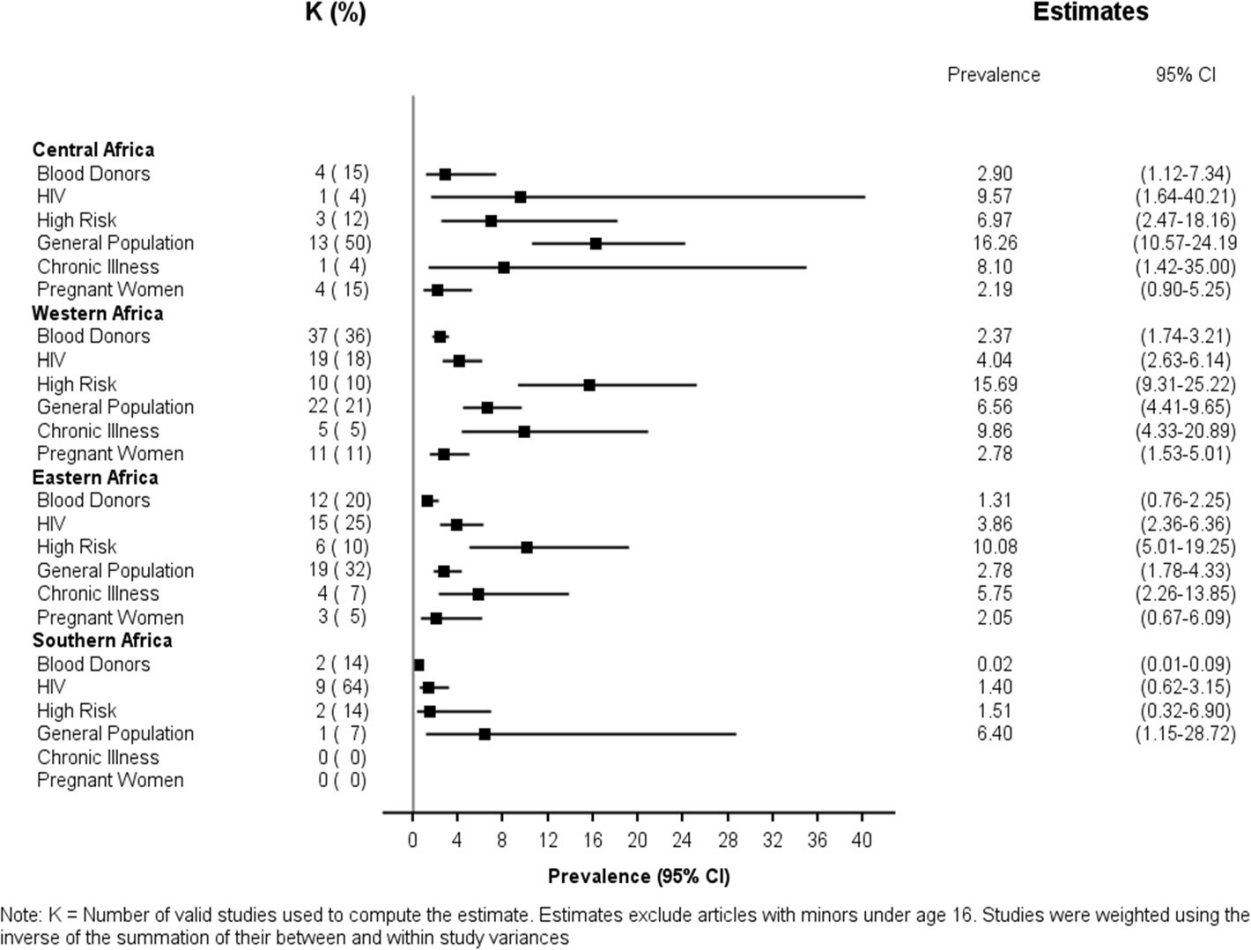
Adjusted multivariable adult HCV prevalence for each cohort by region

Due to the significant interaction of region-by-cohort (*p* = .01), a weighted random-effects multivariable model was used to estimate *adult HCV* prevalences for each cohort within its Sub-Saharan region. Because so few studies had adequate representation of children, and it is likely that the prevalence of HCV among children is different but not adequately captured across studies, we excluded studies that included children for the multi-variable estimates. Figure 3 displays these adult prevalence estimates. Blood donor populations consistently had the lowest adult estimates within each region but varied across regions, with the highest blood donor prevalence in Central and Western Africa (2.90 and 2.37 %, respectively). The lowest adult blood donor HCV prevalence was in Southern Africa with an estimate of 0.02 % (95 % CI: 0.01–0.09 %). Interestingly, outside of the Southern African region, higher adult prevalence estimates were observed among high risk populations, with the highest noted in Western Africa (15.69 %; 95 % CI: 9.31–25.22 %). Across general populations, the highest prevalence was observed in Central Africa and Southern Africa, with adult prevalence estimates of 16.26 and 6.40 %, respectively. However, there was only one study among the general population in South Africa, making any strong conclusions difficult. In contrast, the lowest general adult population estimates were observed in Eastern Africa.

### Overall pooled estimates

While the average population prevalence of Hepatitis C varied significantly across studies (*p* < .001), it was possible to account for sampling error as well as between-studies error using a weighted generalized linear mixed model with random intercepts. Using this approach, the overall prevalence of HCV in SSA, when excluding minors under age 16 was 3.82 % (95 % CI: 3.20–4.55 %); when children were included, the estimate was similar at 3.80 % (95 % CI: 3.20–4.50 %).

We next derived population estimates that accounted for the proportion of adults within each region of SSA (see Additional file 1: Table S1 which provides the adjusted estimates for each region), given that the number of adults in each SSA region varies. These adjusted estimates project 21,315,010 adult cases in SSA, with the greatest proportions occurring in Western and Central Africa. The overall meta-analysis projected 3.82 % of adult cases across SSA; conversely, the prevalence estimate weighted by the proportion of adults within each region provided a prevalence estimate of 3.94 %.

## Discussion

**Fig. 4:**
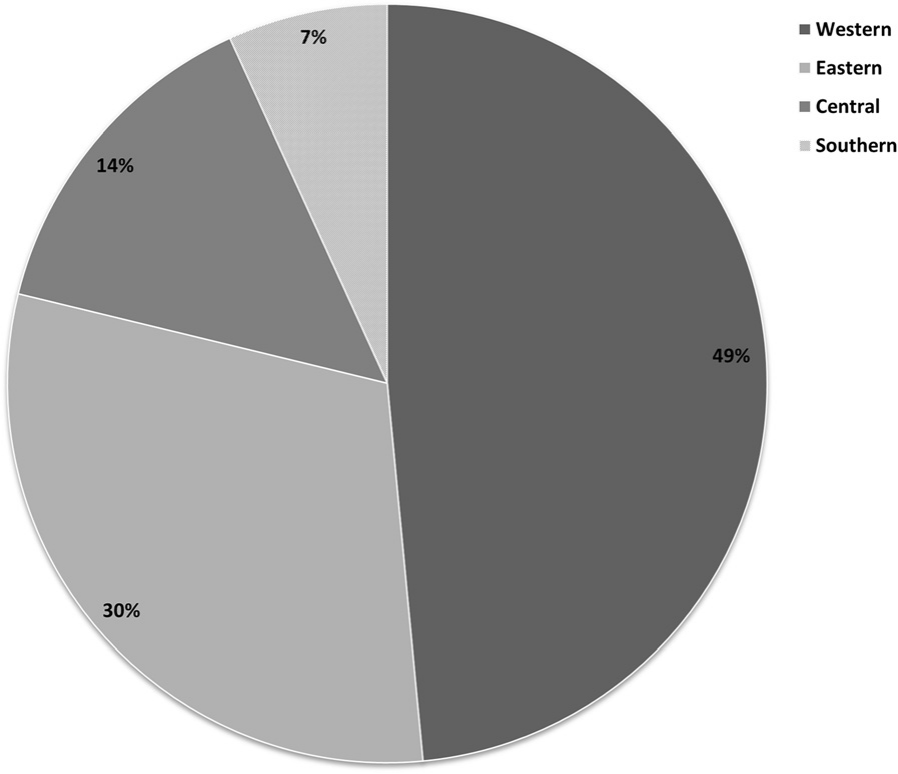
Proportion of studies included in meta-analyses by Region

**Fig. 5:**
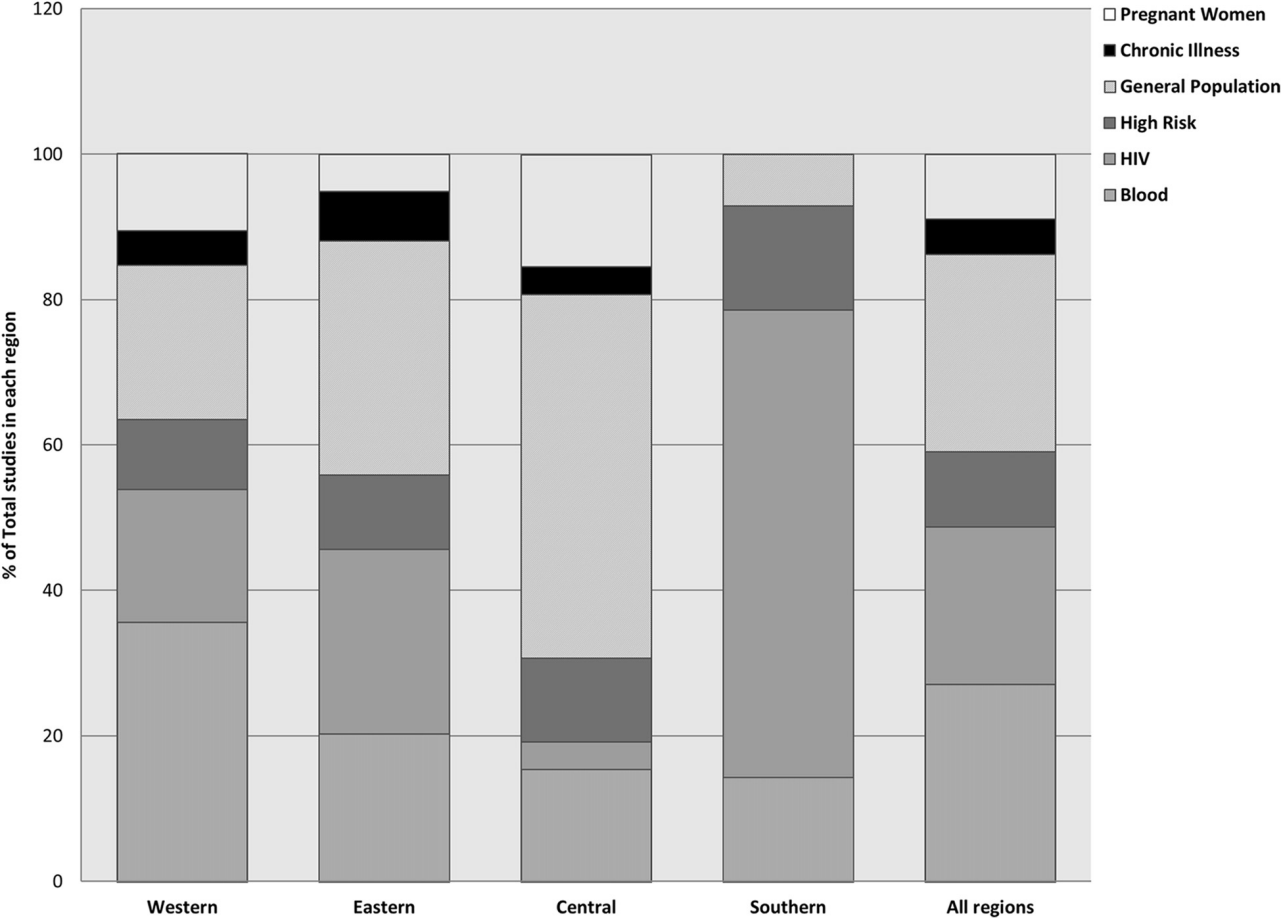
Proportion of studies included in meta-analyses by cohort and region

In this study, we examined the sero-prevalence of HCV across Sub-Saharan Africa. Several key findings resulted from this study. First and foremost, we found large variation among studies that have reported on the sero-prevalence of HCV across SSA and found that the distribution of cohorts across regions did not accurately represent SSA as a whole (Figs. 4 and 5). Nearly half of all studies were conducted in the western region, and the largest proportions of studies occurred among blood donors and the general population. The distribution of cohorts represented in each region was also highly varied. Further, too few studies report prevalence estimates among children, limiting the production of reliable estimates of the sero-prevalence among children.

Secondly, similar to the study by Rao et al. [5], we found significant variation in HCV prevalence across SSA regions as well as population cohorts. The highest prevalence estimates were observed in Central and Western Africa, with the lowest prevalence noted in Southern Africa. Blood donors consistently had the lowest sero-prevalence estimates. Studies among pregnant women generally produced lower estimates compared to general population and high risk cohorts. We tested whether there were significant differences in HCV prevalence by publication year, diagnostic assay, or whether children were included in studies and found no significant variation among these potential modifiers.

Combining this high heterogeneity in the representation of regions and cohorts in published studies, with the noted significant variations in sero-prevalence by both region and cohort, has important implications. First, it significantly limits the reliability and interpretation of pooled sero-prevalence estimates, and questions the usefulness of pooled estimates when describing the HCV epidemics across SSA. Standard meta-analysis pooled estimates weight by the number of studies and do not readily account for the distribution of the overall sampling population. Blood donor cohort studies make up over a quarter of all published studies and blood donor derived studies had the lowest sero-prevalence estimates at 1.78 % overall, with significant variation by region. Blood donors are typically younger, more often urban, and male, and prior studies have shown that they are not always representative of the overall population [14–16]. In a pooled meta-analysis approach, however, these estimates carry a significant weight due to the number of studies and may provide falsely low estimates. Studies from Western Africa represent almost half of all studies, and sero-prevalence estimates from this region are significantly lower than Eastern Africa, and higher than Southern Africa estimates. A meta-analysis derived pooled estimate does not importantly capture this variation.

Further, comparing these estimates across regions is profoundly limited due to the heterogeneity of cohorts represented across regions. For example, Central Africa had the highest uni-variable sero-prevalence at 7.82 %. However, 50 % of studies in Central Africa were derived from general population cohorts that exhibited a high sero-prevalence of 16.26 %. In Eastern Africa, Western Africa, and Southern Africa, general population cohorts made up 32, 21 and 7 % of the studies in their region, respectively. One approach would be to adjust or weight for the proportion of the sampling population that comprises each cohort in each region. However, there are no standard populations or accurate estimates identifying the proportion of the overall population comprising each cohort. At this time, the only plausible weighting would be to consider the population sizes within each region from known census data. Among adults, our meta-analysis derived sero-prevalence estimate was 3.82 %. When we weighted the meta-analysis prevalence estimates by census data, thus accounting for the number of adults in each region, the pooled sero-prevalence estimate increased to 3.94 %. We believe that extreme caution should be used when interpreting the pooled and weighted estimates, both because of the heterogeneity in the representation of the cohorts as well as the varied estimates among cohorts. However, it does illustrate that weighting estimates can lead to different, and perhaps, more accurate pooled estimates. In the case of SSA, the inability to weight or adjust for cohort proportions in and across regions limits the interpretation and accuracy of the estimates.

It is also important to highlight that our pooled estimate of 3.82 % was among adults only, due to the limited reported data among children. Adults make up 52 % of the overall population in SSA [13]. Thus, the total number of HCV cases in SSA is likely higher. Children, however, are likely to have lower HCV sero-prevalence estimates. Thus, an overall sero-prevalence, that includes children, would likely be lower than the pooled adult prevalence estimate. Studies that sample among the youth are profoundly lacking but are sorely needed to help understand the HCV epidemic across SSA. While there is likely an age cohort effect, such that the risk of HCV increases with age as has been seen in other parts of the world, previous work also shows a bi-modal prevalence peak in younger populations, especially in Western Africa [3]. Only the conduct of population-based sampling will allow us an appropriate understanding of the HCV burden among children across SSA.

Another key point derived from this study is the need for careful consideration of cohort classification. We observed significantly lower sero-prevalence among blood donors when compared to the general populations. Rao et al. combined the general population with blood donors in an overall low risk cohort [5]. This study emphasizes the need to distinguish estimates from blood donors and the general population. Further, pregnant women generally have lower sero-prevalence estimates than the general population, and thus should be viewed as a separate cohort. Defining high and low risk cohorts for HCV risk in SSA is generally difficult, largely due to the limited data on past and present transmission pathways and risks associated with HCV infection. More research is needed to accurately and consistently define appropriate cohorts when considering HCV prevalence in SSA.

As with all studies, there are limitations to our study. First, our study is externally limited by the lack of representation across regions and cohorts, as well as among children. Our multivariable meta-analysis findings (Fig. 3) excluded children and focus on estimates across cohorts and regions only among adults. Moreover, the lack of available information on age distributions of the cohorts studied make age-adjusted prevalence estimates, even among the adult population, unattainable and unreliable. Finally, we do not account for frequency of active, viremic infection, due to limited data on this variable.

## Conclusions

This meta-analysis offers a timely update of the burgeoning HCV disease burden in SSA. Importantly, this report highlights the stark differences in cohort representation across regions among published reports, and emphasizes the variability of HCV sero-prevalence by both region and cohort. A large proportion of the global HCV burden occurs in SSA, necessitating the need for more representative studies on the HCV epidemic across SSA.

## Abbreviations

HCV, Hepatitis C Virus; SSA, Sub-Saharan Africa; UNAIDS, The Joint United Nations Programme on HIV/AIDS; WHO, World Health Organization.

## Additional file

Additional file 1: Adult infection estimates and references for prevalence estimates. Table S1 provides HCV infection estimates in Sub-Saharan Africa among individuals ≥ 15 years old per region. All studies used in the main paper to estimate prevalence are additionally referenced. (DOCX 39 kb)
