# Supplementary material for: A Synthesis of Hepatitis C prevalence estimates in Sub-Saharan Africa: 2000–2013
Source: BMC Infect Dis. 2016 Jun 13;16:283. doi: 10.1186/s12879-016-1584-1 (PMC4906983; doi:10.1186/s12879-016-1584-1)
Supplement: Additional file 1: — Adult infection estimates and references for prevalence estimates. Table S1 provides HCV infection estimates in Sub-Saharan Africa among individuals ≥ 15 years old per region. All studies used in the main paper to estimate prevalence are additionally referenced. (DOCX 39 kb) [file 12879_2016_1584_MOESM1_ESM.docx]

Supplementary Appendix

**Additional file 1: Table S1**

*HCV Infection estimates in Sub-Saharan Africa in ≥ 15 years old per region.*

| ***African Region*** | ***N Pop WHO data*** | ***Proportion of Population from each SSA Region*** | ***Estimated HCV Prevalence per Region (from Table 1)*** | ***Adjusted estimated N of HCV infected adults*** |
| --- | --- | --- | --- | --- |
| *Central* | *79,280,089.00* | *0.15* | *7.82%* | *6,199,702.96* |
| *West* | *197,082,039.60* | *0.36* | *4.14%* | *8,159,196.44* |
| *East* | *221,715,242.20* | *0.41* | *3.00%* | *6,651,457.27* |
| *Southern* | *42,312,928.13* | *0.08* | *0.72%* | *304,653.08* |
| *Total* | *540,390,298.93* |  |  | *21,315,009.75* |
| *** UN population estimates for 2015 in Sub-Saharan Africa for the ≥15 years. Representing 56.2% of the overall population.* | | | | |

**The Proportion of Adult population within each Cohort is based on literature research. * Ref17, ** Ref 18,20,33, *** Ref 19, ^ Calculated as (1 – Σ (N in the other cohorts per region)).**

**References used in the analysis**

1. Abreha, T., et al., *Genotypes and viral load of hepatitis C virus among persons attending a voluntary counseling and testing center in Ethiopia.* J Med Virol, 2011. **83**(5): p. 776-82.

2. Acquaye, J.K. and D. Tettey-Donkor, *Frequency of hepatitis C virus antibodies and elevated serum alanine transaminase levels in Ghanaian blood donors.* West Afr J Med, 2000. **19**(4): p. 239-41.

3. Adegoke, O.A., et al., *Seroprevalence of hepatitis C virus infection in Nigerians with type 2 diabetes mellitus.* Niger J Clin Pract, 2008. **11**(3): p. 199-201.

4. Adewole, O.O., et al., *Hepatitis B and C virus co-infection in Nigerian patients with HIV infection.* J Infect Dev Ctries, 2009. **3**(5): p. 369-75.

5. Adjei, A.A., et al., *Correlates of HIV, HBV, HCV and syphilis infections among prison inmates and officers in Ghana: A national multicenter study.* BMC Infect Dis, 2008. **8**: p. 33.

6. Adjei, A.A., et al., *Prevalence of human immunodeficiency virus, hepatitis B virus, hepatitis C virus and syphilis among prison inmates and officers at Nsawam and Accra, Ghana.* J Med Microbiol, 2006. **55**(Pt 5): p. 593-7.

7. Adjei, A.A., et al., *Correlates of hepatitis C virus infection among incarcerated Ghanaians: a national multicentre study.* J Med Microbiol, 2007. **56**(Pt 3): p. 391-7.

8. Adoga, M.P., et al., *Human immunonodeficiency virus, hepatitis B virus and hepatitis C virus: sero-prevalence, co-infection and risk factors among prison inmates in Nasarawa State, Nigeria.* J Infect Dev Ctries, 2009. **3**(7): p. 539-47.

9. Agasa, B., et al., *Prevalence of sickle cell disease in a northeastern region of the Democratic Republic of Congo: what impact on transfusion policy?* Transfus Med, 2010. **20**(1): p. 62-5.

10. Ajayi, E.A.A., A.O.; Adegun, P.T., Ajayi, I.A., *Baseline CD4+ T lymphocyte cell count, hepatitis B and C viruses seropositivity in adults with Human Immunodeficiency virus infection at tertiary hospital in Nigeria.* Pan African Medical Journal, 2011. **9**(6).

11. Akinbami, A.A., et al., *Seroprevalence of hepatitis C infection in HIV patients using a rapid one-step test strip kit.* Nig Q J Hosp Med, 2010. **20**(3): p. 144-6.

12. Alao, O.O., E.; Araoye, M., *The Sero-prevalecne of Hepatitis C virus (Hcv) infection among prospective blood donors in a Nigerian Tertiary Health Institution.* The Internet Journal of Epidemiology, 2008. **7**(2).

13. Ali, S., et al., *Association of Hepatitis C Virus Infection with Type II Diabetes in Ethiopia: A Hospital-Based Case-Control Study.* Interdiscip Perspect Infect Dis, 2012. **2012**: p. 354656.

14. Allain, J.P., et al., *Transfusion-transmitted infectious diseases.* Biologicals, 2009. **37**(2): p. 71-7.

15. Alli J.A.; Okonko, I.O.A., O.A., *A serosurvey of blood parasites (Plasmodium, Microfilaria, HIV, HBsAG, HCV antibodies) in propective Nigerian Blood donors.* Research Journal of Medical Sciences, 2010. **4**(4): p. 255-275.

16. Amadi E.S.; Ononiwu, C.E., *The epidemiology of Hepatitis C virus infection among patients attending the federal ddental clinic, Enugu.* Trends in Medical Reseach, 2009. **4**(4): p. 91-95.

17. Amin, J., et al., *HIV and hepatitis C coinfection within the CAESAR study.* HIV Med, 2004. **5**(3): p. 174-9.

18. Ampofo, W., et al., *Prevalence of blood-borne infectious diseases in blood donors in Ghana.* J Clin Microbiol, 2002. **40**(9): p. 3523-5.

19. Apea-kubi K.A.;Yamaguchi, S., *HTLV-1 and other viral sexually transmitted infections in antenatal and ginaecological patients in Ghana.* WAJM, 2006. **25**(1): p. 17-21.

20. Atina, J.O., et al., *Prevalence of hepatitis A, B, C and human immunodeficiency virus seropositivity among patients with acute icteric hepatitis at the Kenyatta National Hospital, Nairobi.* East Afr Med J, 2004. **81**(4): p. 183-7.

21. Ayele, W., et al., *Higher prevalence of anti-HCV antibodies among HIV-positive compared to HIV-negative inhabitants of Addis Ababa, Ethiopia.* J Med Virol, 2002. **68**(1): p. 12-7.

22. Ayolabi, C.I., ;Taiwo, M.A., *Sero-prevalence of hepatitis C virus among blood donors in Lagos, Nigeria.* African Journal of Biotechnology, 2006. **5**(20): p. 1944-1946.

23. Baby, M., et al., *[Prevalence and risk factors of hepatitis C virus infection in chronic hemodialysis patients at the University Hospital of Point G, Bamako, Mali].* Mali Med, 2011. **26**(2): p. 12-5.

24. Balogun, T.M., I. Akinsete, and M.A. Durosinmi, *Risk factors and seroprevalence of hepatitis C virus antibody among blood donors in Lagos.* Niger Postgrad Med J, 2012. **19**(1): p. 36-9.

25. Balogun, T.M., S. Emmanuel, and E.F. Ojerinde, *HIV, Hepatitis B and C viruses' coinfection among patients in a Nigerian tertiary hospital.* Pan Afr Med J, 2012. **12**: p. 100.

26. Balogun, W.O., et al., *Low prevalence of hepatitis-C viral seropositivity among patients with type-2 diabetes mellitus in a tertiary hospital.* J Natl Med Assoc, 2006. **98**(11): p. 1805-8.

27. Barth, R.E., et al., *Presence of occult HBV, but near absence of active HBV and HCV infections in people infected with HIV in rural South Africa.* J Med Virol, 2011. **83**(6): p. 929-34.

28. Bassey, E.B.M., A.E.; Udo, S.M.; Umo, A.N., *Parallel and overlapping Human Immunodeficiency Virus, Hepatitis B and C virus infections among pregnant women in the Federal Capital territory, Abuja, Nigeria.* Online J Health Allied Sicences, 2009. **8**(1): p. 1-4.

29. Berhe, N., B. Myrvang, and S.G. Gundersen, *Intensity of Schistosoma mansoni, hepatitis B, age, and sex predict levels of hepatic periportal thickening/fibrosis (PPT/F): a large-scale community-based study in Ethiopia.* Am J Trop Med Hyg, 2007. **77**(6): p. 1079-86.

30. Biggar, R.J., et al., *Hepatitis C virus genotype 4 in Ugandan children and their mothers.* Emerg Infect Dis, 2006. **12**(9): p. 1440-3.

31. Blanton, R.E.S., E.A., *Population-based differences in Schistosoma mansoni- and Hepatitis C- induced disease.* The Journal of Infectious Diseases, 2002. **185**.

32. Bowring, A.L., et al., *An urgent need to scale-up injecting drug harm reduction services in Tanzania: prevalence of blood-borne viruses among drug users in Temeke District, Dar-es-Salaam, 2011.* Int J Drug Policy, 2013. **24**(1): p. 78-81.

33. Buseri, F.I., M.A. Muhibi, and Z.A. Jeremiah, *Sero-epidemiology of transfusion-transmissible infectious diseases among blood donors in Osogbo, south-west Nigeria.* Blood Transfus, 2009. **7**(4): p. 293-9.

34. Candotti, D., et al., *Serological and molecular screening for viruses in blood donors from Ntcheu, Malawi: high prevalence of HIV-1 subtype C and of markers of hepatitis B and C viruses.* J Med Virol, 2001. **65**(1): p. 1-5.

35. Candotti, D., F. Sarkodie, and J.P. Allain, *Residual risk of transfusion in Ghana.* Br J Haematol, 2001. **113**(1): p. 37-9.

36. Candotti, D., et al., *Frequent recovery and broad genotype 2 diversity characterize hepatitis C virus infection in Ghana, West Africa.* J Virol, 2003. **77**(14): p. 7914-23.

37. Cantaloube, J.F., et al., *Analysis of hepatitis C virus strains circulating in Republic of the Congo.* J Med Virol, 2010. **82**(4): p. 562-7.

38. Chasela, C.S., et al., *Hepatitis B virus infection among HIV-infected pregnant women in Malawi and transmission to infants.* J Hepatol, 2014. **60**(3): p. 508-14.

39. Collenberg, E., et al., *Seroprevalence of six different viruses among pregnant women and blood donors in rural and urban Burkina Faso: A comparative analysis.* J Med Virol, 2006. **78**(5): p. 683-92.

40. Combe, P., et al., *Hepatitis B and C infections, human immunodeficiency virus and other sexually transmitted infections among women of childbearing age in Cote d'Ivoire, West Africa.* Trans R Soc Trop Med Hyg, 2001. **95**(5): p. 493-6.

41. Croce, F., et al., *Risk factors for HIV/AIDS in a low HIV prevalence site of sub-Saharan Africa.* Trop Med Int Health, 2007. **12**(9): p. 1011-7.

42. Cunha, L., et al., *Use of replacement blood donors to study the epidemiology of major blood-borne viruses in the general population of Maputo, Mozambique.* J Med Virol, 2007. **79**(12): p. 1832-40.

43. Dahoma, M., et al., *HIV and related risk behavior among men who have sex with men in Zanzibar, Tanzania: results of a behavioral surveillance survey.* AIDS Behav, 2011. **15**(1): p. 186-92.

44. de Waal, N., et al., *Mass needle stick injury in children from the Western cape.* J Trop Pediatr, 2006. **52**(3): p. 192-6.

45. Dieye, T.N., et al., *[Seroprevalence of hepatitis C virus (HCV) in Senegalease blood donors].* Dakar Med, 2006. **51**(1): p. 47-52.

46. Diop, S., et al., *[Prevention of transfusion transmitted malaria in endemic area].* Transfus Clin Biol, 2009. **16**(5-6): p. 454-9.

47. Diop-Ndiaye, H., et al., *Hepatitis B, C seroprevalence and delta viruses in HIV-1 Senegalese patients at HAART initiation (retrospective study).* J Med Virol, 2008. **80**(8): p. 1332-6.

48. Diouf, M.L., et al., *[Prevalence of hepatitis B and C viruses in a chronic hemodialysis center in Dakar].* Dakar Med, 2000. **45**(1): p. 1-4.

49. Dokekias, A.E., et al., *[Seroprevalence of viral hepatitis C in polytransfused patients at Central University Hospital of Brazzaville].* Bull Soc Pathol Exot, 2003. **96**(4): p. 279-82.

50. Dray, X., et al., *[Prevalences of HIV, hepatitis B and hepatitis C in blood donors in the Republic of Djibouti].* Med Trop (Mars), 2005. **65**(1): p. 39-42.

51. Duru, M.U., H.S. Aluyi, and K.C. Anukam, *Rapid screening for co-infection of HIV and HCV in pregnant women in Benin City, Edo State, Nigeria.* Afr Health Sci, 2009. **9**(3): p. 137-42.

52. Egah, D.Z., et al., *Hepatitis B surface antigen, hepatitis C and HIV antibodies in a low-risk blood donor group, Nigeria.* East Mediterr Health J, 2007. **13**(4): p. 961-6.

53. Ejele, O.A., C.A. Nwauche, and O. Erhabor, *Seroprevalence of hepatitis C virus in the Niger Delta of Nigeria.* Niger Postgrad Med J, 2006. **13**(2): p. 103-6.

54. Ejiofor, O.S., et al., *The role of blood transfusion on the prevalence of hepatitis C virus antibodies in children with sickle cell anaemia in Enugu, South East Nigeria.* Niger J Clin Pract, 2009. **12**(4): p. 355-8.

55. Eller, M.A., Eller, L.A., *Single-cell level response of HIV-specific and cytomegalovirus-specific CD4 T cells correlate with viral control in chronic HIV-1 subtype A infection.* J Acquir Immune Defic Syndr Hum Retrovirol, 2012. **61**(1): p. 9 -18.

56. Erhabor, O., O.A. Ejele, and C.A. Nwauche, *The risk of transfusion-acquired hepatitis-C virus infection among blood donors in Port Harcourt: the question of blood safety in Nigeria.* Niger J Clin Pract, 2006. **9**(1): p. 18-21.

57. Etard, J.F., et al., *Hepatitis C antibodies among blood donors, Senegal, 2001.* Emerg Infect Dis, 2003. **9**(11): p. 1492-3.

58. Fang, C.T., et al., *Human immunodeficiency virus-1 and hepatitis C virus RNA among South African blood donors: estimation of residual transfusion risk and yield of nucleic acid testing.* Vox Sang, 2003. **85**(1): p. 9-19.

59. Fasola, F.A., T.R. Kotila, and J.O. Akinyemi, *Trends in transfusion-transmitted viral infections from 2001 to 2006 in Ibadan, Nigeria.* Intervirology, 2008. **51**(6): p. 427-31.

60. Fessehaye, N., D. Naik, and T. Fessehaye, *Transfusion transmitted infections - a retrospective analysis from the National Blood Transfusion Service in Eritrea.* Pan Afr Med J, 2011. **9**: p. 40.

61. Forbi, J., et al., *Serological markers and risk factors for hepatitis B and hepatitis C viruses among students in a Nigerian university.* East Afr J Public Health, 2009. **6**(2): p. 152-5.

62. Forbi, J.C., et al., *The role of triple infection with hepatitis B virus, hepatitis C virus, and human immunodeficiency virus (HIV) type-1 on CD4+ lymphocyte levels in the highly HIV infected population of North-Central Nigeria.* Mem Inst Oswaldo Cruz, 2007. **102**(4): p. 535-7.

63. Forbi, J.C., et al., *Urban-rural estimation of hepatitis C virus infection sero-prevalence in north Central Nigeria.* East Afr J Public Health, 2010. **7**(4): p. 367-8.

64. Foupouapouognigni, Y., et al., *Hepatitis B and C virus infections in the three Pygmy groups in Cameroon.* J Clin Microbiol, 2011. **49**(2): p. 737-40.

65. Franzeck, F.C., et al., *Viral hepatitis and rapid diagnostic test based screening for HBsAg in HIV-infected patients in rural Tanzania.* PLoS One, 2013. **8**(3): p. e58468.

66. Fritzsche, C., et al., *Hepatitis B and C: neglected diseases among health care workers in Cameroon.* Trans R Soc Trop Med Hyg, 2013. **107**(3): p. 158-64.

67. Gededzha, M.P.M.M.J., *Should routine serological sceenirng for HCV be mandatory in HIV/AIDS patients enrolling for HAART in South Africa?* SAMJ, 2010. **100**(12): p. 814-815.

68. Guimaraes Nebenzahl, H., et al., *Prevalence of human immunodeficiency virus, hepatitis C virus, hepatitis B virus and syphilis among individuals attending anonymous testing for HIV in Luanda, Angola.* S Afr Med J, 2013. **103**(3): p. 186-8.

69. Halim, N.K. and O.I. Ajayi, *Risk factors and seroprevalence of hepatitis C antibody in blood donors in Nigeria.* East Afr Med J, 2000. **77**(8): p. 410-2.

70. Halim, N.K., et al., *Hepatitis B surface antigen and antibody to hepatitis C virus among accident and emergency patients.* East Afr Med J, 2001. **78**(9): p. 480-3.

71. Harania, R.S., et al., *HIV, hepatitis B and hepatitis C coinfection in Kenya.* AIDS, 2008. **22**(10): p. 1221-2.

72. Hassall, O.W., et al., *The microbiologic safety of umbilical cord blood transfusion for children with severe anemia in Mombasa, Kenya.* Transfusion, 2012. **52**(7): p. 1542-51.

73. Hladik, W., et al., *Prevalence and screening costs of hepatitis C virus among Ugandan blood donors.* Trop Med Int Health, 2006. **11**(6): p. 951-4.

74. Hoffmann, C.J., et al., *Prevalence and associations with hepatitis B and hepatitis C infection among HIV-infected adults in South Africa.* Int J STD AIDS, 2012. **23**(10): p. e10-3.

75. Imarengiaye, C.O., et al., *Risk of transfusion-transmitted hepatitis C virus in a tertiary hospital in Nigeria.* Public Health, 2006. **120**(3): p. 274-8.

76. Inyama, P.U.U., C.J., *Prevalence of antibodies to Hepatitis C virus among Nigerian patients with HIV infection.* Online J Health Allied, 2005. **4**(2).

77. Jeremiah, Z.A., et al., *Prevalence of antibodies to hepatitis C virus in apparently healthy Port Harcourt blood donors and association with blood groups and other risk indicators.* Blood Transfus, 2008. **6**(3): p. 150-5.

78. Jobarteh, M., et al., *Seroprevalence of hepatitis B and C virus in HIV-1 and HIV-2 infected Gambians.* Virol J, 2010. **7**: p. 230.

79. Kabinda, J.M. and B.P. Katchunga, *Les hépatites virales B et C chez les porteurs du virus de l’immunodéficience humaine à Bukavu (Sud-Kivu), République démocratique du Congo.* Journal Africain d'Hépato-Gastroentérologie, 2010. **4**(4): p. 230-235.

80. Kallestrup, P., et al., *Low prevalence of hepatitis C virus antibodies in HIV-endemic area of Zimbabwe support sexual transmission as the major route of HIV transmission in Africa.* AIDS, 2003. **17**(9): p. 1400-2.

81. Kania, D., et al., *A new strategy to improve the cost-effectiveness of human immunodeficiency virus, hepatitis B virus, hepatitis C virus, and syphilis testing of blood donations in sub-Saharan Africa: a pilot study in Burkina Faso.* Transfusion, 2009. **49**(10): p. 2237-40.

82. Kapembwa, K.C., et al., *HIV, Hepatitis B, and Hepatitis C in Zambia.* J Glob Infect Dis, 2011. **3**(3): p. 269-74.

83. Karuru, J.W., et al., *Prevalence of HCV and HCV/HIV co-infection among in-patients at the Kenyatta National Hospital.* East Afr Med J, 2005. **82**(4): p. 170-2.

84. Kitundu, J., et al., *Post-transfusion hepatitis C seroprevalence in Tanzanian children.* Ann Trop Paediatr, 2001. **21**(4): p. 343-8.

85. Koate, B.B., F.I. Buseri, and Z.A. Jeremiah, *Seroprevalence of hepatitis C virus among blood donors in Rivers State, Nigeria.* Transfus Med, 2005. **15**(5): p. 449-51.

86. Kubio, C., et al., *Blood transfusion practice in a rural hospital in Northern Ghana, Damongo, West Gonja District.* Transfusion, 2012. **52**(10): p. 2161-6.

87. Kurbanov, F., et al., *A new subtype (subgenotype) Ac (A3) of hepatitis B virus and recombination between genotypes A and E in Cameroon.* J Gen Virol, 2005. **86**(Pt 7): p. 2047-56.

88. Ladep, N.G., et al., *Rates and impact of hepatitis on human immunodeficiency virus infection in a large African cohort.* World J Gastroenterol, 2013. **19**(10): p. 1602-10.

89. Lassey, A.T., et al., *Hepatitis C virus seroprevalence among mothers delivering at the Korle-Bu Teaching Hospital, Ghana.* East Afr Med J, 2004. **81**(4): p. 198-201.

90. Laurent, C., et al., *HIV and hepatitis C virus coinfection, Cameroon.* Emerg Infect Dis, 2007. **13**(3): p. 514-6.

91. Laurent, C., et al., *Seroepidemiological survey of hepatitis C virus among commercial sex workers and pregnant women in Kinshasa, Democratic Republic of Congo.* Int J Epidemiol, 2001. **30**(4): p. 872-7.

92. Lesi, O.A. and M.O. Kehinde, *Hepatitis C virus infection in patients with sickle cell anaemia at the Lagos University Hospital.* Niger Postgrad Med J, 2003. **10**(2): p. 79-83.

93. Lesi, O.A., et al., *Hepatitis B and C virus infection in Nigerian patients with HIV/AIDS.* Niger Postgrad Med J, 2007. **14**(2): p. 129-33.

94. Lodenyo, H., et al., *Hepatitis B and C virus infections and liver function in AIDS patients at Chris Hani Baragwanath Hospital, Johannesburg.* East Afr Med J, 2000. **77**(1): p. 13-5.

95. Mabayoje, V.O.A., P.O.; Opaleye, O.O., *Prevalence of Hepatitis B surface antigen, hepatitis C and Human immunodefciency virus antiboidies in a population of students of tertiary institution in Nigeria.* African Journal of Clinical and Experimental Microbiology, 2010. **11**(2): p. 68-74.

96. Madzime, S., et al., *Seroprevalence of hepatitis C virus infection among indigent urban pregnant women in Zimbabwe.* Cent Afr J Med, 2000. **46**(1): p. 1-4.

97. Maida, M.J., et al., *Prevalence of hepatitis C infection in Malawi and lack of association with sexually transmitted diseases.* Eur J Epidemiol, 2000. **16**(12): p. 1183-4.

98. Matee, M.I., P.M. Magesa, and E.F. Lyamuya, *Seroprevalence of human immunodeficiency virus, hepatitis B and C viruses and syphilis infections among blood donors at the Muhimbili National Hospital in Dar es Salaam, Tanzania.* BMC Public Health, 2006. **6**: p. 21.

99. Mayaphi, S.H., Rossouw, T.M., *HBV/HIV co-infection: The dynamics of HBV in South African patietns with AIDS.* S Afr Med J, 2012. **102**(3): p. 157-162.

100. Mbanya, D.N., D. Takam, and P.M. Ndumbe, *Serological findings amongst first-time blood donors in Yaounde, Cameroon: is safe donation a reality or a myth?* Transfus Med, 2003. **13**(5): p. 267-73.

101. Mbanya, D.N. and C. Tayou, *Blood safety begins with safe donations: update among blood donors in Yaounde, Cameroon.* Transfus Med, 2005. **15**(5): p. 395-9.

102. Mboto, C.I., et al., *Prevalence, sociodemographic characteristics and risk factors for hepatitis C infection among pregnant women in Calabar municipality, Nigeria.* Hepat Mon, 2010. **10**(2): p. 116-20.

103. Mboto, C.I., et al., *Hepatitis C antibodies in asymptomatic first-time blood donors in The Gambia: prevalence and risk factors.* Br J Biomed Sci, 2005. **62**(2): p. 89-91.

104. Mboto, C.I., et al., *Prevalence of HIV-1, HIV-2, hepatitis C and co-infection in The Gambia.* West Afr J Med, 2009. **28**(1): p. 16-9.

105. Mboto, C.I.A., B.E.; Lennox, J and Lawson, U.D., *Hepatitis C virus prevalence rate and risk factors in jaundice and non-jaundice in-patients seen in two tertiary health facilities in South Nigeria.* Journal of Microbiology and Biotechnology Research, 2013. **3**(4): p. 1-6.

106. Meschi, S., et al., *The prevalence of antibodies to human herpesvirus 8 and hepatitis B virus in patients in two hospitals in Tanzania.* J Med Virol, 2010. **82**(9): p. 1569-75.

107. Mogtomo, M.L., et al., *[Screening of infectious microorganisms in blood banks in Douala (1995-2004)].* Sante, 2009. **19**(1): p. 3-8.

108. Moore, E., et al., *Favourable one-year ART outcomes in adult Malawians with hepatitis B and C co-infection.* J Infect, 2010. **61**(2): p. 155-63.

109. Mosendane, T.K., M.C., *Nurses at risk for ocupationally acquired blood-borne virus infection at South African academic hospital.* S Afr Med J, 2012. **102**(3): p. 153-156.

110. Msuya, S.E., et al., *Seroprevalence of hepatitis B and C viruses among women of childbearing age in Moshi Urban, Tanzania.* East Afr Med J, 2006. **83**(2): p. 91-4.

111. Muasya, T., et al., *Prevalence of hepatitis C virus and its genotypes among a cohort of drug users in Kenya.* East Afr Med J, 2008. **85**(7): p. 318-25.

112. Nagalo, B.M., et al., *Seroprevalence and incidence of transfusion-transmitted infectious diseases among blood donors from regional blood transfusion centres in Burkina Faso, West Africa.* Trop Med Int Health, 2012. **17**(2): p. 247-53.

113. Nagalo, M.B., et al., *Seroprevalence of human immunodeficiency virus, hepatitis B and C viruses and syphilis among blood donors in Koudougou (Burkina Faso) in 2009.* Blood Transfus, 2011. **9**(4): p. 419-24.

114. Nagu, T.J., M. Bakari, and M. Matee, *Hepatitis A, B and C viral co-infections among HIV-infected adults presenting for care and treatment at Muhimbili National Hospital in Dar es Salaam, Tanzania.* BMC Public Health, 2008. **8**: p. 416.

115. Naniche, D., et al., *Alterations in T cell subsets in human immunodeficiency virus-infected adults with co-infections in southern Mozambique.* Am J Trop Med Hyg, 2011. **85**(4): p. 776-81.

116. Ndako, J.A., et al., *Occurrence of hepatitis C virus infection in type 2 diabetic patients attending Plateau state specialist hospital Jos Nigeria.* Virol J, 2009. **6**: p. 98.

117. Ndjomou, J., et al., *Hepatitis C virus infection and genotypes among human immunodeficiency virus high-risk groups in Cameroon.* J Med Virol, 2002. **66**(2): p. 179-86.

118. Ndong-Atome, G.R., et al., *Hepatitis C virus prevalence and genetic diversity among pregnant women in Gabon, central Africa.* BMC Infect Dis, 2008. **8**: p. 82.

119. Ndong-Atome, G.R., et al., *High prevalence of hepatitis C virus infection and predominance of genotype 4 in rural Gabon.* J Med Virol, 2008. **80**(9): p. 1581-7.

120. Ndong-Atome, G.R., et al., *Absence of intrafamilial transmission of hepatitis C virus and low risk for sexual transmission in rural central Africa indicate a cohort effect.* J Clin Virol, 2009. **45**(4): p. 349-53.

121. Nerrienet, E., et al., *Hepatitis C virus infection in cameroon: A cohort-effect.* J Med Virol, 2005. **76**(2): p. 208-14.

122. Njouom, R., et al., *Phylogeography, risk factors and genetic history of hepatitis C virus in Gabon, central Africa.* PLoS One, 2012. **7**(8): p. e42002.

123. Njouom, R., et al., *Predominance of hepatitis C virus genotype 4 infection and rapid transmission between 1935 and 1965 in the Central African Republic.* J Gen Virol, 2009. **90**(Pt 10): p. 2452-6.

124. Njouom, R., et al., *Transmission of hepatitis C virus among spouses in Cameroon and the Central African Republic.* J Med Virol, 2011. **83**(12): p. 2113-8.

125. Njouom, R., et al., *High rate of hepatitis C virus infection and predominance of genotype 4 among elderly inhabitants of a remote village of the rain forest of South Cameroon.* J Med Virol, 2003. **71**(2): p. 219-25.

126. Njouom, R., et al., *Hepatitis C virus infection among pregnant women in Yaounde, Cameroon: prevalence, viremia, and genotypes.* J Med Virol, 2003. **69**(3): p. 384-90.

127. Njouom, R., et al., *Low risk of mother-to-child transmission of hepatitis C virus in Yaounde, Cameroon: the ANRS 1262 study.* Am J Trop Med Hyg, 2005. **73**(2): p. 460-6.

128. Nkrumah, B., et al., *Hepatitis B and C viral infections among blood donors from rural Ghana.* Ghana Med J, 2011. **45**(3): p. 97-100.

129. Ntagirabiri, R., F. Ngendakumana, and T. Niyongabo, *Co-infection par le virus de l’immunodéficience humaine et le virus de l’hépatite C au Burundi.* Journal Africain d'Hépato-Gastroentérologie, 2012. **6**(2): p. 128-131.

130. Nur, Y.A., et al., *Prevalence of serum antibodies against bloodborne and sexually transmitted agents in selected groups in Somalia.* Epidemiol Infect, 2000. **124**(1): p. 137-41.

131. Nwankiti, O.O., et al., *Hepatitis C Virus infection in apparentenly healthy individuals with family history of diabetes in Vom, Plateau State Nigeria.* Virol J, 2009. **6**: p. 110.

132. Nwankwo, E.M., I., *Seroprevalence of major blood-borne infections among blood donors in Kano, Nigeria.* Turk J Med Sci, 2012. **42**(2): p. 337- 341.

133. Nwokediuko, S.C. and J.M. Oli, *Hepatitis C virus infection in Nigerians with diabetes mellitus.* Niger J Clin Pract, 2008. **11**(2): p. 94-9.

134. Nyirenda, M., et al., *Prevalence of infection with hepatitis B and C virus and coinfection with HIV in medical inpatients in Malawi.* J Infect, 2008. **57**(1): p. 72-7.

135. Obienu, O., et al., *Risk factors for hepatitis C virus transmission obscure in nigerian patients.* Gastroenterol Res Pract, 2011. **2011**: p. 939673.

136. Obuseh, F.A., et al., *Aflatoxin B1 albumin adducts in plasma and aflatoxin M1 in urine are associated with plasma concentrations of vitamins A and E.* Int J Vitam Nutr Res, 2010. **80**(6): p. 355-68.

137. Ogunro, P.S., et al., *Prevalence of anti-hepatitis C virus antibodies in pregnant women and their offspring in a tertiary hospital in Southwestern Nigeria.* J Infect Dev Ctries, 2007. **1**(3): p. 333-6.

138. Oje, O.J., W.F. Sule, and D. Famurewa, *Dual positivity of hepatitis B surface antigen and anti-hepatitis C virus antibody and associated factors among apparently healthy patients of Ekiti State, Nigeria.* Viral Immunol, 2012. **25**(6): p. 448-55.

139. Ola, S.O.O., J.A., *Serum Hepatitis C virus and hepatitis B surface antigenaemia in Nigerian patients with acute icteric hepatitis.* WAJM, 2002. **21**(3): p. 215- 217.

140. Ola, S.O.O., J.A., *Occult HBV infection among a coohort of Nigerian adults.* J Infect Dev Ctries, 2009. **3**(6): p. 442-446.

141. Olokoba, A.B., et al., *Risk factors and clinical presentation of hepatitis C virus infection in Nigerians with chronic liver disease.* Trop Doct, 2011. **41**(3): p. 146-7.

142. Onakewhor, J.U. and F.E. Okonofua, *Seroprevalence of Hepatitis C viral antibodies in pregnancy in a tertiary health facility in Nigeria.* Niger J Clin Pract, 2009. **12**(1): p. 65-73.

143. Opaleye, O.O., et al., *HBV, HCV co-infection among blood donors in Nigeria.* Indian J Pathol Microbiol, 2010. **53**(1): p. 182-3.

144. O'Reilly, J.I., et al., *Risk Factors and Seroprevalence of Hepatitis C among Patients Hospitalized at Mulago Hospital, Uganda.* J Trop Med, 2011. **2011**: p. 598341.

145. Otedo, A.E., et al., *Seroprevalence of hepatitis B and C in maintenance dialysis in a public hospital in a developing country.* S Afr Med J, 2003. **93**(5): p. 380-4.

146. Otegbayo, J.A., et al., *Prevalence of hepatitis B and C seropositivity in a Nigerian cohort of HIV-infected patients.* Ann Hepatol, 2008. **7**(2): p. 152-6.

147. Ouedraogo, A.S., et al., *[Prevalence of anti-CMV antibodies in blood donors in Ouagadougou (Burkina Faso)].* Med Sante Trop, 2012. **22**(1): p. 107-9.

148. Owusu-Ofori, S., et al., *Predonation screening of blood donors with rapid tests: implementation and efficacy of a novel approach to blood safety in resource-poor settings.* Transfusion, 2005. **45**(2): p. 133-40.

149. Ozsazuwa, F.O., O.V., *Sero-Epidemiology of Human immunodeficency virus, hepatitis B and C among pregnant women in rural communities of Abji area council, Nigeria.* TAF Prev Med Bull, 2012. **11**(4): p. 431- 438.

150. Parboosing, R., I. Paruk, and U.G. Lalloo, *Hepatitis C virus seropositivity in a South African Cohort of HIV co-infected, ARV naive patients is associated with renal insufficiency and increased mortality.* J Med Virol, 2008. **80**(9): p. 1530-6.

151. Pasquier, C., et al., *Distribution and heterogeneity of hepatitis C genotypes in hepatitis patients in Cameroon.* J Med Virol, 2005. **77**(3): p. 390-8.

152. Patel, P., et al., *Prevalence of hepatitis B and hepatitis C coinfections in an adult HIV centre population in Gaborone, Botswana.* Am J Trop Med Hyg, 2011. **85**(2): p. 390-4.

153. Pepin, J., et al., *Iatrogenic transmission of human T cell lymphotropic virus type 1 and hepatitis C virus through parenteral treatment and chemoprophylaxis of sleeping sickness in colonial Equatorial Africa.* Clin Infect Dis, 2010. **51**(7): p. 777-84.

154. Pepin, J., et al., *Risk factors for hepatitis C virus transmission in colonial Cameroon.* Clin Infect Dis, 2010. **51**(7): p. 768-76.

155. Pirillo, M.F., et al., *Seroprevalence of hepatitis B and C viruses among HIV-infected pregnant women in Uganda and Rwanda.* J Med Virol, 2007. **79**(12): p. 1797-801.

156. Plamondon, M., et al., *Hepatitis C virus infection in Guinea-Bissau: a sexually transmitted genotype 2 with parenteral amplification?* PLoS One, 2007. **2**(4): p. e372.

157. Puato, M., et al., *Does HCV infection have a more favourable outcome in Tanzanian people? Data from the Lugalawa study.* Dig Liver Dis, 2007. **39**(9): p. 891-2.

158. Rabenau, H.F., et al., *Prevalence- and gender-specific immune response to opportunistic infections in HIV-infected patients in Lesotho.* Sex Transm Dis, 2010. **37**(7): p. 454-9.

159. Ramarokoto, C.E., et al., *Seroprevalence of hepatitis C and associated risk factors in urban areas of Antananarivo, Madagascar.* BMC Infect Dis, 2008. **8**: p. 25.

160. Ramos, J.M., et al., *Prevalence of HIV, HBV, HCV, HTLV and Treponema pallidum among patients attending a rural hospital in Southern Ethiopia.* J Clin Virol, 2012. **53**(3): p. 268-9.

161. Randriamanantany, Z.A.R., D.H. , *Prevalence and trend of hepatitis C virus among blood donors in Antananarivo, from 2003 to 2009.* Transfusion Clinique et Biologique: Journal de la Societe Fracaise de Transfusion Sanguine, 2012. **19**(2): p. 52-56.

162. Rouet, F., et al., *HBV and HCV prevalence and viraemia in HIV-positive and HIV-negative pregnant women in Abidjan, Cote d'Ivoire: the ANRS 1236 study.* J Med Virol, 2004. **74**(1): p. 34-40.

163. Sadoh, A.E.S., W.E., *HIV co-infection with Hepatits B and C viruses among Nigerian children in antiretroviral treatment programme.* SAJCH, 2011. **5**(1): p. 7-10.

164. Sagoe, K.W., et al., *Prevalence and impact of hepatitis B and C virus co-infections in antiretroviral treatment naive patients with HIV infection at a major treatment center in Ghana.* J Med Virol, 2012. **84**(1): p. 6-10.

165. Sarkodie, F., et al., *Screening for viral markers in volunteer and replacement blood donors in West Africa.* Vox Sang, 2001. **80**(3): p. 142-7.

166. Segbena, A.Y., et al., *[Human immunodeficiency virus, hepatitis C virus and hepatitis B viruses in patients with sickle-cell disease in Togo].* Transfus Clin Biol, 2005. **12**(6): p. 423-6.

167. Seremba, E., et al., *Poor performance of hepatitis C antibody tests in hospital patients in Uganda.* J Med Virol, 2010. **82**(8): p. 1371-8.

168. Serme, A.K., et al., *[Prevalence of Hepatitis C virus infection in pregnant women and mother-child transmission in Ouagadougou, Burkina Faso].* Bull Soc Pathol Exot, 2006. **99**(2): p. 108-9.

169. Simpore, J., et al., *Toxoplasma gondii, HCV, and HBV seroprevalence and co-infection among HIV-positive and -negative pregnant women in Burkina Faso.* J Med Virol, 2006. **78**(6): p. 730-3.

170. Stark, K., et al., *Seroepidemiology of TT virus, GBC-C/HGV, and hepatitis viruses B, C, and E among women in a rural area of Tanzania.* J Med Virol, 2000. **62**(4): p. 524-30.

171. Stevens, W., et al., *Baseline morbidity in 2,990 adult African volunteers recruited to characterize laboratory reference intervals for future HIV vaccine clinical trials.* PLoS One, 2008. **3**(4): p. e2043.

172. Sutcliffe, S., et al., *HIV-1 prevalence and herpes simplex virus 2, hepatitis C virus, and hepatitis B virus infections among male workers at a sugar estate in Malawi.* J Acquir Immune Defic Syndr, 2002. **31**(1): p. 90-7.

173. Telatela, S.P., M.I. Matee, and E.K. Munubhi, *Seroprevalence of hepatitis B and C viral co-infections among children infected with human immunodeficiency virus attending the paediatric HIV care and treatment center at Muhimbili National Hospital in Dar-es-Salaam, Tanzania.* BMC Public Health, 2007. **7**: p. 338.

174. Tess, B.H., et al., *Seroprevalence of hepatitis C virus in the general population of northwest Tanzania.* Am J Trop Med Hyg, 2000. **62**(1): p. 138-41.

175. Tessema, B., et al., *Seroprevalence of HIV, HBV, HCV and syphilis infections among blood donors at Gondar University Teaching Hospital, Northwest Ethiopia: declining trends over a period of five years.* BMC Infect Dis, 2010. **10**: p. 111.

176. Tremeau-Bravard, A.O., I.C.; Ticao C.J.; Abubakar, J.J., *Seroprevalence of Hepatitis B and C among HIV-positive population in Abuja, Nigeria.* Afr Health Sci, 2012. **12**(3): p. 312-317.

177. Udeze, O.O., O.I., Donbraye, E.; Suel, W.F.; Fadeyi, A.; Unche, L.N., *Seroprevalence of Hepatitis C antibodies amongst blood donors in Ibadan, Southwestern, Nigeria.* World Applied Sciences Journal, 2009. **7**(8): p. 1023-1028.

178. Ugbebor, O., et al., *The prevalence of hepatitis B and C viral infections among pregnant women.* N Am J Med Sci, 2011. **3**(5): p. 238-41.

179. Ukonu, A.B. and U. Augustine, *The prevalence of hepatitis C Virus (HCV) among lichen planus patients and its clinical pattern at the University of Abuja Teaching Hospital, Gwagwalada, Abuja, Nigeria.* Glob J Health Sci, 2012. **4**(5): p. 113-9.

180. Vardas, E., et al., *Viral hepatitis in South African healthcare workers at increased risk of occupational exposure to blood-borne viruses.* J Hosp Infect, 2002. **50**(1): p. 6-12.

181. Vermeulen, M., et al., *Impact of individual-donation nucleic acid testing on risk of human immunodeficiency virus, hepatitis B virus, and hepatitis C virus transmission by blood transfusion in South Africa.* Transfusion, 2009. **49**(6): p. 1115-25.

182. Vray, M., et al., *Molecular epidemiology of hepatitis B virus in Dakar, Senegal.* J Med Virol, 2006. **78**(3): p. 329-34.

183. Walusansa, V. and M. Kagimu, *Screening for hepatitis C among HIV positive patients at Mulago hospital in Uganda.* Afr Health Sci, 2009. **9**(3): p. 143-6.

184. Wester, C.W., et al., *Serological evidence of HIV-associated infection among HIV-1-infected adults in Botswana.* Clin Infect Dis, 2006. **43**(12): p. 1612-5.

185. Zeba, M.T., et al., *HCV prevalence and co-infection with HIV among pregnant women in Saint Camille Medical Centre, Ouagadougou.* Trop Med Int Health, 2011. **16**(11): p. 1392-6.
